# Oxidative Stress and Antioxidant Responses of *Phormidium ambiguum* and *Microcystis aeruginosa* Under Diurnally Varying Light Conditions

**DOI:** 10.3390/microorganisms8060890

**Published:** 2020-06-12

**Authors:** Guligena Muhetaer, Senavirathna M. D. H. Jayasanka, Takeshi Fujino

**Affiliations:** Graduate School of Science and Engineering, Saitama University, 255 Shimo-Okubo, Sakura-ku, Saitama 338-8570, Japan; gurigina.m.775@ms.saitama-u.ac.jp (G.M.); fujino@mail.saitama-u.ac.jp (T.F.)

**Keywords:** harmful cyanobacteria, hydrogen peroxide, species characteristics, linear relationships, photo stress

## Abstract

Two harmful cyanobacteria species (*Phormidium ambiguum* and *Microcystis aeruginosa*) were exposed to diurnal light-intensity variation to investigate their favorable and stressed phases during a single day. The photosynthetically active radiation (PAR) started at 0 µmol·m^−2^·s^−1^ (06:00 h), increased by ~25 µmol·m^−2^·s^−1^ or ~50 µmol·m^−2^·s^−1^ every 30 min, peaking at 300 µmol·m^−2^·s^−1^ or 600 µmol·m^−2^·s^−1^ (12:00 h), and then decreased to 0 µmol·m^−2^·s^−1^ (by 18:00 h). The H_2_O_2_ and antioxidant activities were paralleled to light intensity. Higher H_2_O_2_ and antioxidant levels (guaiacol peroxidase, catalase (CAT), and superoxidase dismutase) were observed at 600 µmol·m^−2^·s^−1^ rather than at 300 µmol·m^−2^·s^−1^. Changes in antioxidant levels under each light condition differed between the species. Significant correlations were observed between antioxidant activities and H_2_O_2_ contents for both species, except for the CAT activity of *P. ambiguum* at 300 µmol·m^−2^·s^−1^. Under each of the conditions, both species responded proportionately to oxidative stress. Even under maximum light intensities (300 µmol·m^−2^·s^−1^ or 600 µmol·m^−2^·s^−1^ PAR intensity), neither species was stressed. Studies using extended exposure durations are warranted to better understand the growth performance and long-term physiological responses of both species.

## 1. Introduction

The growth and spread of cyanobacteria have increased, thus threatening today′s water bodies and supplies worldwide [1,2]. Global warming and abundant nutrition supply have promoted the spread of cyanobacteria, which, among others, generate bad odors by producing substances such as 2-methylisoborneol, releasing cyanotoxins and forming blooms, thus making many water bodies unusable [3,4,5]. In addition, some cyanobacterial species can produce allelochemicals that are harmful to other aquatic species [6,7,8]. Therefore, numerous studies have focused on suppressing or preventing their growth, globally [9,10,11].

During cyanobacteria control efforts, chemical control measures are discouraged due to their potentially harmful secondary effects on ecosystems [12,13,14], while non-chemical methods require knowledge of the interactions of cyanobacteria with the natural environment, their responses to changing environmental factors or stresses, and their interaction with other species (allopathy). Currently, this approach is being extensively studied by various research groups [15,16,17,18,19,20,21]. In addition, many studies have focused on the physiology and morphology of cyanobacteria under natural and laboratory-derived conditions [22,23]. However, despite those findings, knowledge gaps remain to be filled.

Biological, chemical, and physical factors collectively determine the occurrence and distribution of cyanobacteria in the environment [24,25,26]. Physical factors, such as temperature and light, influence the growth and distribution of cyanobacteria [27,28]. To establish a presence in an ecosystem, cyanobacteria require temperature and light-intensity conditions within suitable ranges, which can vary between cyanobacterial species [27,29,30]. Under preferable environmental conditions for most cyanobacteria, such as tropical and subtropical conditions, temperature factors do not show significant diurnal variation [31]. However, the light conditions certainly change, regardless of the geological location. In natural ecosystems, the sunlight typically peaks at approximately noon-hour and then gradually decreases, following that point into the evening. This light cycle also affects the physiological conditions of cyanobacteria in a diurnal manner [32], and extreme light conditions (high or low) can be disadvantageous for cyanobacteria.

The photosynthetic species produce reactive oxygen species (ROS) as a byproduct of the photosynthesis process, which is harmful when accumulated in cells [33]. Therefore, a balance between ROS generation and antioxidant activities is required to maintain cell homeostasis [34]. Unfavorable conditions, including excess light, can disturb this balance, leading to oxidative stress [35,36]. When the solar eradiation is varying diurnally, the production of ROS in cyanobacteria then varies, and the antioxidants balance should therefore be adjusted accordingly. Both ROS production and antioxidant activities of cyanobacteria under diurnally varying light conditions have yet to be fully elucidated. This will deepen our understanding of the diurnal variation of cyanobacteria physiology and helps to determine the sufficient time scale to apply cyanobacteria control measures, such as bubbling and mixing which light is the primary factor [37].

In this study, the oxidative stress (H_2_O_2_) and antioxidant (guaiacol peroxidase (GPX), catalase (CAT), ascorbic peroxidase (APX), and superoxidase dismutase (SOD)) responses of cyanobacteria to diurnal changes in the light intensity were studied. Two photosynthetically active radiation (PAR) levels, 300 and 600 µmol·m^−2^·s^−1^, were selected as maximum light intensities, and the responses of two cyanobacterial species, *Phormidium ambiguum* and *Microcystis aeruginosa*, were tested for gradually varying light conditions. *P. ambiguum* is a non-heterocystous filamentous cyanobacterial species, categorized as a benthic cyanobacterium [38], while *M. aeruginosa* is a floating (buoyant) and colony foaming type cyanobacterium [39]. Both cyanobacterial species used in this study are known for their harmful environmental effects, due to their cytotoxin release, as well as their increasing growth in tropical and subtropical water bodies [40,41].

## 2. Materials and Methods

### 2.1. Cyanobacteria Cultures and Incubation

*P. ambiguum* (strain NIES 2119) and *M. aeruginosa* (strain NIES 111) were obtained from the National Institute for Environmental Studies, Tsukuba, Japan. Both species were cultured for 14 days at 20 °C under a 12 h:12 h light:dark cycle inside an incubator (MIR-254, Sanyo, Tokyo, Japan). Light was provided with cool white fluorescent lamps and the intensity was maintained at 20–30 µmol·m^−2^·s^−1^ PAR. The nutrient medium was 100% BG-11 [42]. During the incubation period, each culture was manually shaken three times every day during the light phase.

### 2.2. Experimental Setup and Procedure

Following the 14-day incubation, 3 replicate conical flasks (500 mL Pyrex clear glass conical flasks) from each of the *P. ambiguum* and *M. aeruginosa* cyanobacteria cultures were made, maintaining the 0.6 ± 0.02 optical density measured at 730 nm (OD_730_) using a spectrophotometer (UVmini-1240, Shimadzu, Kyoto, Japan). The dilution of the cyanobacteria culture was accomplished with BG11 nutrient medium. In all experiments, the temperature was maintained at 20 °C in an incubator, whereas the lighting conditions changed from 0 µmol·m^−2^·s^−1^ (at 06:00 h) to 300 µmol·m^−2^·s^−1^ or 600 µmol·m^−2^·s^−1^ (at 12:00 h) by changing the lighting intensity by ~25 µmol·m^−2^·s^−1^ or ~50 µmol·m^−2^·s^−1^ every 30 min with a VBP-L24-C2 light (Valore, Kyoto, Japan). The light intensity was then decreased at the same rate (until 18:00 h). The lighting condition was controlled with warm light-emitting diode panel lights, and the light intensity was measured using a quantum flux meter (Apogee, MQ-200, Logan, UT, USA). Cyanobacteria samples from each flask were collected for analysis every 3 h, at 06:00 h, 09:00 h, 12:00 h, 15:00 h, 18:00 h, and 21:00 h. To facilitate mixing, each flask was manually shaken at the time of sampling.

### 2.3. H_2_O_2_ Concentration

Cellular H_2_O_2_ contents were estimated according to standard methods [43]. Briefly, 1 mL was collected from each flask and the supernatants were removed by centrifugation at 10,000× *g* for 10 min at 4 °C. The cell pellets were washed once with ultrapure water (Milli-Q direct 5, Merck KGaA, Darmstadt, Germany). To extract cellular H_2_O_2_, cell pellets were homogenized in 1 mL of 0.1 M pH 6.5 phosphate buffer and centrifuged at 10,000× *g* for 10 min at 4 °C. A total of 750 μL of 1% titanium chloride in 20% H_2_SO_4_ (v/v) was then added to initiate the reaction. The optical absorption was measured at 410 nm using a spectrophotometer (UVmini-1240), following centrifugation (10,000× *g* for 5 min) at room temperature (25 ± 2 °C). The H_2_O_2_ concentration was determined using a standard curve, prepared using a series of samples with known H_2_O_2_ concentration.

### 2.4. GPX-Activity Assay

The GPX activity was assayed as described by Hoda et al. [44] and MacAdam et al. [45], with modifications. Cyanobacteria cells were harvested by centrifuging 1 mL samples at 10,000× *g* at 4 °C for 10 min and removing the supernatant and cell pellets, which were homogenized in 1 mL potassium phosphate buffer (100 mM, pH 7.0). A total of 65 µl of enzyme extract was then mixed with 920 μL of potassium phosphate buffer (100 mM, pH 7) containing 20 mM guaiacol. With the addition of 15 μL of 0.6% H_2_O_2_, the reaction was then started, and the absorbance change was recorded at 470 nm every 10 s for 3 min using UV mini-1240. GPX activity was calculated using an extinction coefficient of 26.6 mM/cm.

### 2.5. CAT-Activity Assay

CAT activity was measured using the method described by Aebi [46]. A total of 1 mL of each culture was centrifuged at 10,000× *g* at 4 °C for 10 min. The supernatant was removed, and the cell pellets were homogenized in 1 mL potassium phosphate buffer (50 mM, pH 7.0), containing 0.1 mM EDTA. After centrifuging again (10,000× *g* at 4 °C for 10 min), the supernatant was collected as the enzyme extract. The CAT activity was measured by reacting 15 µL of 750 mM H_2_O_2_, 920 µL of potassium phosphate buffer, and 65 µL of extract supernatant. Optical absorption was measured at 240 nm using UV mini-1240. The measurements were recorded every 10 s for 3 min, and the CAT activity was calculated using an extinction coefficient of 39.4 mM/cm.

### 2.6. APX-Activity Assay

APX activity was assayed, as described by Nakano and Asada [47]. The decrease in absorbance at 290 nm was recorded every 10 s for 3 min using UV mini-1240. Each reaction mixture was performed in a 1-mL volume. Initially, 920 µL of 50 mM phosphate buffer (pH 7.0), containing 5 mM EDTA, was mixed with 15 µL of 0.5 mM ascorbic acid. Each reaction was then started routinely by adding 15 µL of 1 mM H_2_O_2_. Calculations were performed using a molar extinction coefficient for ascorbate of 2.8 mM/cm.

### 2.7. SOD-Activity Assay

SOD activities were determined by performing nitro blue tetrazolium (NBT) assays, as described by Ewing and Janero [48]. Each sample was mixed with 10 µL of 750 µM NBT, 10 µL of 130 mM methionine, 70 µL of 50 mM phosphate buffer with 100 µM EDTA (pH 7.8), and 10 µL of 20 µM riboflavin solution. The reactions were carried out for 5 min, and the absorbances were recorded at 560 nm using UV mini-1240. Blank reactions were prepared by substituting the sample with an equal volume of 50 mM phosphate buffer (pH 7.8). One unit of SOD activity was defined as the amount of SOD that inhibited the rate of formazan production by 50% at 25 °C.

### 2.8. Data Analysis

One-way analysis of variance (ANOVA), followed by Tukey’s post-hoc test, was performed to test the statistical significance of variations among the means of sample groups. Data were normalized relative to the starting group (06:00 h), by dividing the results of each group by the corresponding 06:00 h group for each replicate. Significant differences between experimental groups of *P. ambiguum* and *M. aeruginosa* were evaluated using a Student’s t-test, assuming equality of variance. Pearson’s correlation analysis was used to evaluate correlations between parameters. Statistical analyses were performed by using IBM SPSS Statistics for Windows, Version 25.0. (IBM Corp, Armonk, NY, USA).

## 3. Results

**Figure 1:**
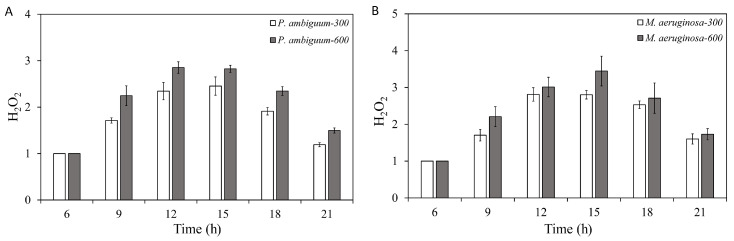
Diurnal variations in the H_2_O_2_ contents of *Phormidium. ambiguum* (strain NIES 2119) (**A**) and *Microcystis aeruginosa* (strain NIES 111) (**B**). The numbers 300 and 600 represent the maximum photosynthetically active radiation (PAR) intensities (in µmol·m^−2^·s^−1^) for two different treatment conditions, where the maximum PAR intensity was reached at 12:00 h. The error bars represent the standard deviations.

The H_2_O_2_ contents of *P. ambiguum* and *M. aeruginosa* increased with increasing light intensity and peaked between 12:00 h and 15:00 h. The H_2_O_2_ content decreased thereafter in parallel with decreasing light intensity. The cyanobacteria were exposed to dark at 18:00 h; however, even at 21:00 h, the H_2_O_2_ contents did not reach the initial level measured at 06:00 h (Figure 1). For *P. ambiguum* exposed to 300 µmol·m^−2^·s^−1^ max PAR intensity, ANOVA testing grouped H_2_O_2_ contents at 12:00 h and 15:00 h and the rest of the time, points were grouped individually (*p* < 0.01, *F* = 97.839). ANOVA testing of *P. ambiguum* exposed to 600 µmol·m^−2^·s^−1^ max PAR intensity grouped H_2_O_2_ contents at 06:00 h, 09:00 h, and 18:00 h, and 12:00 h, 15:00 h, and 21:00 h (*p* < 0.01, *F* = 167.265). For *M. aeruginosa* exposed to 300 µmol·m^−2^·s^−1^ max PAR intensity, ANOVA testing grouped H_2_O_2_ contents at 06:00 h, 09:00 h, and 21:00 h, 12:00 h and 15:00 h, and 18:00 h (*p* < 0.01, *F* = 182. 714). ANOVA testing of *M. aeruginosa* exposed to 600 µmol·m^−2^·s^−1^ max PAR intensity grouped H_2_O_2_ contents at each time point (*p* < 0.01, *F* = 106.817). Comparing the H_2_O_2_ contents of 300 µmol·m^−2^·s^−1^ and 600 µmol·m^−2^·s^−1^, max PAR intensity groups showed that the H_2_O_2_ contents of both *P. ambiguum* and *M. aeruginosa* differed significantly at each time point (*p* < 0.01 for each time point).

**Figure 2:**
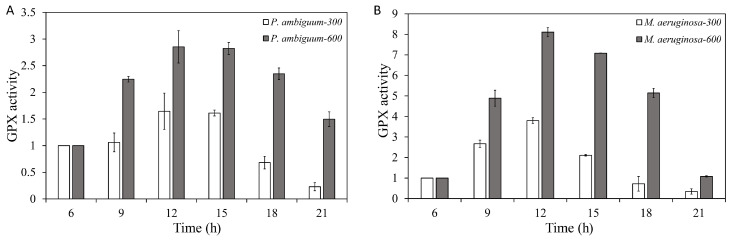
Diurnal variations in the guaiacol peroxidase (GPX) activities of *P. ambiguum* (strain NIES 2119) (**A**) and *M. aeruginosa* (strain NIES 111) (**B**). The numbers 300 and 600 represent the maximum PAR intensities (in µmol·m^−2^·s^−1^) for two different treatment conditions, where the maximum PAR intensity was reached at 12:00 h. The error bars represent the standard deviations.

The GPX activities of both *P. ambiguum* and *M. aeruginosa* increased over time and reached the maximum at 12:00 h when the maximum PAR intensity was reached (300 µmol·m^−2^·s^−1^ or 600 µmol·m^−2^·s^−1^). With decreasing light intensity, the GPX activities of both species were decreased. However, with the 300 µmol·m^−2^·s^−1^ max PAR intensity group, at 18:00 h and 21:00 h, the GPX activities decreased even further than the starting GPX activity (06:00 h). With the 600 µmol·m^−2^·s^−1^ max PAR-intensity, the GPX activity of both species decreased with decreasing light, but the GPX activity of *P. ambiguum* remained higher than the starting GPX activity, even at 21:00 h. For *M. aeruginosa*, GPX activity of 600 µmol·m^−2^·s^−1^ max PAR intensity reached the starting GPX activity (06:00 h) at 21:00 h (Figure 2). For *P. ambiguum* exposed to 300 µmol·m^−2^·s^−1^ max PAR intensity, ANOVA testing grouped at 06:00, 09:00, and 15:00 h; 12:00, 15:00, and 18:00 h; and 21:00 h (*p* < 0.01, *F* = 16.945). ANOVA testing of the *P. ambiguum* exposed to 600 µmol·m^−2^·s^−1^ max PAR intensity grouped GPX activities at 06:00 h and 09:00 h; 12:00 h and 15:00 h; 18:00 h; and 21:00 h (*p* < 0.01, *F* = 35.562). For *M. aeruginosa* exposed to 300 µmol·m^−2^·s^−1^ max PAR intensity, ANOVA testing groped GPX activities at 06:00 h, 18:00 h, and 21:00 h; 12:00 h; and 09:00 h and 15:00 h (*p* < 0.01, *F* = 18.050). ANOVA testing of *M. aeruginosa* exposed to 600 µmol·m^−2^·s^−1^ max PAR intensity grouped GPX activities at 06:00 h and 21:00 h; 06:00 h, 09:00 h, and 18:00 h; 09:00 h, 15:00 h, and 18:00 h; and 12:00 h (*p* < 0.01, *F* = 13.418). A comparison between 300 µmol·m^−2^·s^−1^ and 600 µmol·m^−2^·s^−1^ max PAR intensity groups showed that the GPX activities of *P. ambiguum* and *M. aeruginosa* differed significantly at each time point (*p* < 0.01 for each time point).

**Figure 3:**
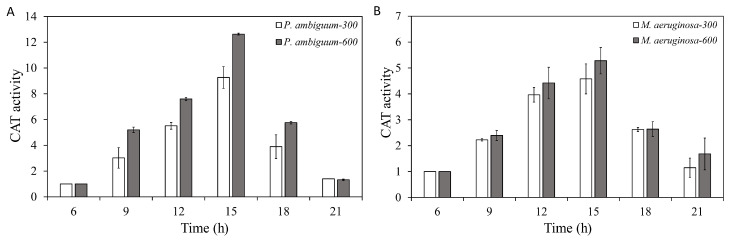
Diurnal variations in the catalase (CAT) activities of *P. ambiguum* (strain NIES 2119) (**A**) and *M. aeruginosa* (strain NIES 111) (**B**). The numbers 300 and 600 represent the maximum PAR intensities (in µmol·m^−2^·s^−1^) for two different treatment conditions, where the maximum PAR intensity was reached at 12:00 h. The error bars represent the standard deviations.

The CAT activities of both *P. ambiguum* and *M. aeruginosa* increased over time but showed a delayed response, as the maximum CAT activities were reached at 15:00 h (which was 3 h after the maximum light intensities of 300 µmol·m^−2^·s^−1^ or 600 µmol·m^−2^·s^−1^ PAR was reached). Decreasing light intensities were paralleled by reduced CAT activities, although the CAT activity did not reach the initial CAT level, even at 21:00 h (Figure 3). For *P. ambiguum* exposed to 300 µmol·m^−2^·s^−1^ max PAR intensity, ANOVA testing grouped CAT activities at 06:00 h, 09:00 h, and 21:00 h; 09:00 h, 18:00 h, and 21:00 h; and 12:00 h and 15:00 h (*p* < 0.01, *F* = 20.489). ANOVA testing of *P. ambiguum* exposed to 600 µmol·m^−2^·s^−1^ max PAR intensity grouped CAT activities at 06:00 h and 21:00 h; 09:00 h, 12:00 h, and 18:00 h; and 15:00 h (*p* < 0.01, *F* = 41.935). For *M. aeruginosa* exposed to 300 µmol·m^−2^·s^−1^ max PAR intensity, ANOVA testing groped CAT activities at 06:00 h and 21:00 h; 9:00 h; 21:00 h, 09:00 h, and 18:00 h; and 12:00 h and 15:00 h (*p* < 0.01, *F* = 24.520). ANOVA testing of *M. aeruginosa* exposed to 600 µmol·m^−2^·s^−1^ max PAR intensity grouped CAT activities at 06:00 h and 21:00 h; 09:00 h and 18:00 h; 12:00 h; and 15:00 h (*p* < 0.01, *F* = 35.619). Comparisons between the 300 µmol·m^−2^·s^−1^ and 600 µmol·m^−2^·s^−1^ max PAR intensity groups showed that the CAT activities of *P. ambiguum* differed significantly at 09:00 h, 12:00 h, 15:00 h, and 18:00 h (*p* < 0.01), although the CAT activities at 06:00 h and 21:00 h were not different (*p* > 0.05). The CAT activities of 300 µmol·m^−2^·s^−1^ and 600 µmol·m^−2^·s^−1^ max PAR intensity groups of *M. aeruginosa* also differed at 09:00 h, 15:00 h, and 21:00 h (*p* < 0.05).

**Figure 4:**
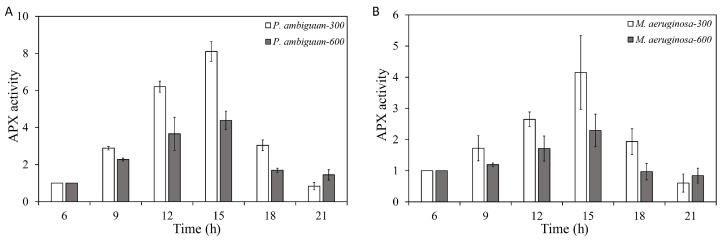
Diurnal variations in the ascorbic peroxidase (APX) activities of *P. ambiguum* (strain NIES 2119) (**A**) and *M. aeruginosa* (strain NIES 111) (**B**). The numbers 300 and 600 represent the maximum PAR intensities (in µmol·m^−2^·s^−1^) for two different treatment conditions, where the maximum PAR intensity was reached at 12:00 h. The error bars represent the standard deviations.

The APX activities of both *P. ambiguum* and *M. aeruginosa* increased with increasing light intensity, but a delayed response was observed, where the maximum APX activity was reached at 15:00 h (3 h after the maximum light intensities were reached). With subsequent decreasing light intensity, the APX activities of both species decreased and reached the initial APX activity (06:00 h) at 21:00 h (Figure 4). For *P. ambiguum* exposed to 300 µmol·m^−2^·s^−1^ max PAR intensity, ANOVA testing grouped APX activities at 06:00 h and 21:00 h; 09:00 h and 18:00 h; and 12:00 h and 18:00 h (*p* < 0.01, *F* = 35.599). ANOVA testing of *P. ambiguum* exposed 600 µmol·m^−2^·s^−1^ max PAR intensity grouped APX activities at 06:00 h, 18:00 h, and 21:00 h; 09:00 h, 18:00 h, and 21:00 h; and 12:00 h and 15:00 h (*p* < 0.01, *F* = 15.069). For *M. aeruginosa* exposed to 300 µmol·m^−2^·s^−1^ max PAR intensity, ANOVA testing grouped APX activities at 06:00 h, 18:00 h, and 21:00 h; 06:00 h, 09:00 h, and 15:00 h; and 12:00 h (*p* < 0.01, *F* = 18.050). ANOVA testing of *M. aeruginosa* exposed to 600 µmol·m^−2^·s^−1^ max PAR intensity grouped APX activities at 06:00 h and 21:00 h; 06:00 h, 09:00 h, and 18:00 h; 09:00 h, 15:00 h, and 18:00 h; and 12:00 h (*p* < 0.01, *F* = 13.418). Comparisons between the 300 µmol·m^−2^·s^−1^ and 600 µmol·m^−2^·s^−1^ max PAR intensity groups showed that the differences in APX activities for both species were significantly higher in the 300 µmol·m^−2^·s^−1^ max PAR intensity groups, from 09:00 h to 18:00 h (*p* < 0.01 for each light condition for both species). The 21:00 APX activities of *P. ambiguum* were significantly lower in the 300 µmol·m^−2^·s^−1^ max PAR intensity group than in the 600 µmol·m^−2^·s^−1^ max PAR intensity group (*p* < 0.01), although no differences were observed for *M. aeruginosa*.

**Figure 5:**
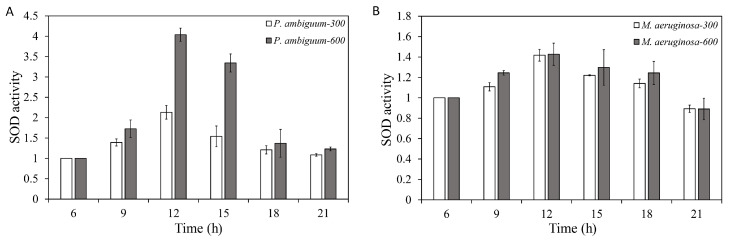
Diurnal variations in the super oxidase dismutase (SOD) activities of *P. ambiguum* (strain NIES 2119) (**A**) and *M. aeruginosa* (strain NIES 111) (**B**). The numbers 300 and 600 represent the maximum PAR intensities (in µmol·m^−2^·s^−1^) for two different treatment conditions, where the maximum PAR intensity was reached at 12:00 h. The error bars represent the standard deviations.

The SOD activities of both *P. ambiguum* and *M. aeruginosa* increased with the light intensity. With decreasing light, SOD activity decreased for both species and approached the starting level at 21:00 h, for both 300 µmol·m^−2^·s^−1^ and 600 µmol·m^−2^·s^−1^ max PAR conditions (Figure 5). For *P. ambiguum* exposed to 300 µmol·m^−2^·s^−1^ max PAR intensity, ANOVA testing grouped SOD activities at 06:00 h, 18:00 h, and 21:00 h; 09:00 h and 15:00 h; and 12:00 h (*p* < 0.01, *F* = 30.725). ANOVA testing of *P. ambiguum* exposed to 600 µmol·m^−2^·s^−1^ max PAR intensity grouped SOD activities at 06:00 h, 18:00 h, and 21:00 h; 09:00 h, 18:00 h, and 21:00 h; 12:00 h; and 15:00 h (*p* < 0.01, *F* = 65.914). For *M. aeruginosa* exposed to 300 µmol·m^−2^·s^−1^ max PAR intensity, ANOVA testing grouped SOD activities at 06:00 h and 21:00 h; 06:00 h and 09:00 h; 09:00 h, 15:00 h, and 18:00 h; and 12:00 h, 15:00 h, and 18:00 h (*p* < 0.01, *F* = 10.859). ANOVA testing of *M. aeruginosa* exposed to 600 µmol·m^−2^·s^−1^ max PAR intensity grouped SOD activities at 06:00 h and 21:00 h; 06:00 h, 09:00 h, 15:00 h, and 18:00 h; and 09:00 h and 12:00 h (*p* < 0.01, *F* = 7.313). Comparisons between the 300 µmol·m^−2^·s^−1^ and 600 µmol·m^−2^·s^−1^ max PAR intensity groups indicated that the SOD activities of *P. ambiguum* differed at 12:00 h and 15:00 h (*p* < 0.01) and that *M. aeruginosa* showed no significant differences between 300 µmol·m^−2^·s^−1^ and 600 µmol·m^−2^·s^−1^ max PAR intensity conditions (*p* > 0.05).

**Figure 6:**
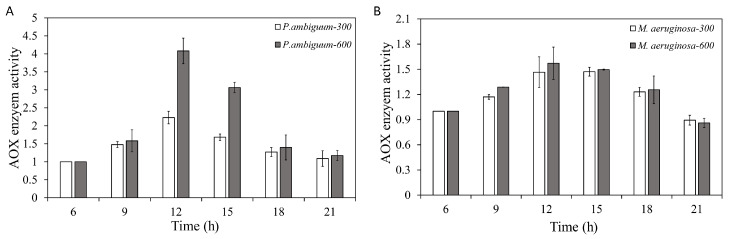
Diurnal variations in the total antioxidant (AOX) activity of *P. ambiguum* (strain NIES 2119) (**A**) and *M. aeruginosa* (strain NIES 111) (**B**). The numbers 300 and 600 represent the maximum PAR intensities (in µmol·m^−2^·s^−1^) for two different treatment conditions, where the maximum PAR intensity was reached at 12:00 h. The error bars represent the standard deviations.

The total antioxidant (AOX) activities of both *P. ambiguum* and *M. aeruginosa* increased with the light intensity. With decreasing light, the AOX activity decreased, and, for both species, the AOX activity approached the starting level at 21:00 h, under both the 300 µmol·m^−2^·s^−1^ and 600 µmol·m^−2^·s^−1^ PAR conditions (Figure 6). For *P. ambiguum* exposed to 300 µmol·m^−2^·s^−1^ max PAR intensity, ANOVA testing grouped AOX activities at 06:00 h and 21:00 h; 18:00 h and 21:00 h; 09:00 h and 15:00 h; and 12:00 h (*p* < 0.01, *F* = 41.711). ANOVA testing of *P. ambiguum* exposed to 600 µmol·m^−2^·s^−1^ max PAR intensity grouped AOX activities at 06:00 h, 18:00 h, and 21:00 h; 09:00 h, 18:00 h, and 21:00 h; 15:00 h; and 12:00 h (*p* < 0.01, *F* = 79.973). For *M. aeruginosa* exposed to 300 µmol·m^−2^·s^−1^ max PAR intensity, ANOVA testing grouped AOX activities at 06:00 h and 21:00 h; 09:00 h and 18:00 h; and 12:00 h and 15:00 h (*p* < 0.01, *F* = 26.143). ANOVA testing of *M. aeruginosa* exposed to 600 µmol·m^−2^·s^−1^ max PAR intensity grouped AOX activities at 06:00 h and 21:00 h; 06:00 h and 18:00 h; 09:00 h, 15:00 h, and 18:00 h; and 12:00 h and 15:00 h (*p* < 0.01, *F* = 12.466). The AOX activities of 300 and 600 µmol·m^−2^·s^−1^ max PAR intensities differed significantly at 12:00 h and 15:00 h, with *P. ambiguum* (*p* < 0.01), while those for *M. aeruginosa* only exhibited a significant difference at 09:00 h (*p* < 0.01). In other cases, no significant differences were observed.

**Figure 7:**
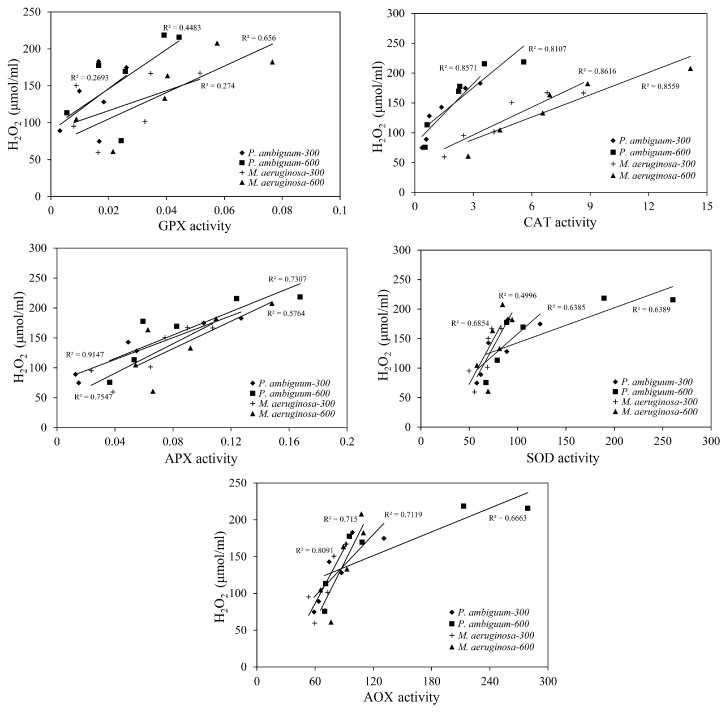
Linear regression relationships between the H_2_O_2_ contents and the antioxidant activities (GPX, CAT, APX, SOD, and AOX) of *P. ambiguum* (strain NIES 2119) and *M. aeruginosa* (strain NIES 111). The numbers 300 and 600 represent the maximum PAR intensities (in µmol·m^−2^·s^−1^) for two different treatment conditions, where the maximum PAR intensity was reached at 12:00 h.

The relationships between the H_2_O_2_ levels and those of the antioxidants (CAT, APX, GPX, and SOD) and the AOX levels were significantly linearly correlated, except for the GPX activity of *P. ambiguum* under the 300 µmol·m^−2^·s^−1^ max PAR intensity (Figure 7). Pearson’s correlation test results are presented in Table 1. Among the antioxidants tested, GPX consistently showed low *R^2^* values and exhibited higher variance. Out of all the relationships, the CAT activity showed the highest *R^2^* values (>0.75), confirming that less variance occurred. When the AOX values were considered, the *R^2^* showed relatively higher values (>0.72).

## 4. Discussion

The H_2_O_2_ contents and the antioxidant activities of *P. ambiguum* and *M. aeruginosa* were highly responsive to the diurnal variations in light intensity. In this study, the only variable factor was the light intensity, where H_2_O_2_ levels were high during times of higher light intensities and decreased at lower light intensities. When cellular H_2_O_2_ level increases, the antioxidant activities correspondingly increase to prevent damage induced by oxidative stress [49,50]. As observed with the H_2_O_2_ levels, the antioxidant activities also varied during the same time frame and followed the H_2_O_2_ levels, which increased at higher light intensities and decreased at lower light intensities. The antioxidant activities of both species were correlated linearly with the H_2_O_2_ contents. Although the H_2_O_2_-antioxidant relationships were varied from strong to weak (depending on the antioxidant species), overall, our findings suggest that the antioxidant levels of both species responded to the cellular H_2_O_2_ level accordingly.

The H_2_O_2_ and antioxidant responses followed the same trends for both maximum light intensity conditions (PAR intensities of 300 µmol·m^−2^·s^−1^ or 600 µmol·m^−2^·s^−1^). Under the maximum PAR intensity of 600 µmol·m^−2^·s^−1^, the cyanobacteria received approximately twice the photon energy of the group with a maximum PAR of 300 µmol·m^−2^·s^−1^. Therefore, it can be anticipated that the experiment groups, which receive higher photon energy, undergo an enhanced rate of photosynthesis. This is evidenced by the increased H_2_O_2_ formed after exposure to a higher light intensity [51,52]. However, at higher light intensities, where the photon energy exceeds tolerable levels for the photosystem, photoinhibition occurs to prevent photodamage [53,54], during which H_2_O_2_ production is reduced with higher light exposure [55,56,57]. As the H_2_O_2_ contents correlated directly with light intensity, even at higher intensities, PAR intensities under 600 µmol·m^−2^·s^−1^ did not subject either cyanobacterial species to photo stress. However, this study only involved a single day diurnal variation, and the H_2_O_2_ levels of the cells did not reach the starting H_2_O_2_ conditions at 06:00 h (even at 21:00 h) for either species. The antioxidant activities almost decreased to the initial conditions by 21:00 h. Therefore, cells may undergo oxidative stress during dark conditions due to the lack of antioxidant activities. If the H_2_O_2_ was continued to be presence in cells, the protein synthesis of photosystems will be inhibited [36] and, in long duration, cell function will be reduced and even cell deaths may occur [58]. Therefore, an extended exposure period is required to better understand the fate of the remaining H_2_O_2_ and adaptation responses.

The antioxidant levels differed between the two species, where the response level was lower for *M. aeruginosa* than *P. ambiguum*, except for GPX. Under high H_2_O_2_ contents, the AOX activity was highly elevated in *P. ambiguum*, but in the dark, both species reached the starting AOX activity level at 21:00 h. This finding suggests that *M. aeruginosa* is less tolerant to oxidative stress than *P. ambiguum* [36,59]. Concerning the correlation between antioxidant responses and H_2_O_2_ contents, both species demonstrated significant linear relationships (with the only exception being for GPX of *P. ambiguum* under a maximum PAR of 300 µmol·m^−2^·s^−1^). Therefore, despite the high AOX content of the *P. ambiguum*, both species were able to maintain balanced antioxidant activity under every light condition of the single day exposure.

The difference in antioxidant levels of the two species can be related to their behavioral characteristics. The *M. aeruginosa* is a buoyant species that floats in a range of depths and might have higher tolerance to oxidative stress [60,61] than benthic *P. ambiguum*. However, in the present study, both species experienced same light intensity variance as the cultures were mixed periodically. The non-different H_2_O_2_ contents between the two species suggested that both species experienced similar levels of oxidative stress. Therefore, less oxidative stress tolerance of *P. ambiguum* triggered the antioxidant activity at a relatively higher rate. Conversely, nonenzymatic antioxidants, primarily carotenoids, protect against ROS in phototrophs, including cyanobacteria [36,62]. The nonenzymatic antioxidants can neutralize ROS prior to triggering the antioxidant enzymes. However, the carotenoid content is reported to be higher in *P. ambiguum* than *M. aeruginosa* [63,64,65]; therefore, it is challenging to determine whether the low antioxidant activity reported in *M. aeruginosa* is due to involvement of nonenzymic antioxidant over the *P. ambiguum*.

Our previous study on the effects of 8 days of exposure to non-varying, high-light intensities (300 µmol·m^−2^·s^−1^ and 600 µmol·m^−2^·s^−1^) confirmed that the OD_730_ and chlorophyll-a contents of cyanobacteria (*Pseudanabaena galeata* and *M. aeruginosa*) were significantly reduced, which was associated with oxidative stress [60]. Although the present research confirmed the relationships between varying oxidative stress and antioxidant responses with light intensity, further investigation into the longer-term effects on the growth and pigmentation of cyanobacteria is warranted. Longer exposure duration will help to better understand the growth performance and physiological responses of cyanobacteria to diurnally varying light conditions. Further, there can be a circadian rhythm in the physiology of cyanobacteria, for which the cellular conditions can be changed diurnally, regardless of the prevailing conditions [66]. In future studies, the circadian rhythm of the cyanobacterial species should also be considered.

## Figures and Tables

**Table 1:** Pearson’s correlation test results of the correlation between H_2_O_2_ and antioxidant levels, i.e., guaiacol peroxidase (GPX), catalase (CAT), ascorbic peroxidase (APX), super oxidase dismutase (SOD), and total antioxidants (AOX).

Condition	Parameter	*R^2^*	*p* Value
*M. aeruginosa*–300 ^1^	SOD	0.780	*p* < 0.01
	APX	0.652	*p* < 0.01
	CAT	0.893	*p* < 0.01
	GPX	0.539	*p* < 0.05
	AOX	0.856	*p* < 0.01
*M. aeruginosa*-600	SOD	0.526	*p* < 0.05
	APX	0.683	*p* < 0.01
	CAT	0.924	*p* < 0.01
	GPX	0.692	*p* < 0.01
	AOX	0.720	*p* < 0.01
*P. ambiguum*-300	SOD	0.748	*p* < 0.01
	APX	0.962	*p* < 0.01
	CAT	0.824	*p* < 0.01
	GPX	0.383	*p* > 0.05
	AOX	0.803	*p* < 0.01
*P. ambiguum*-600	SOD	0.784	*p* < 0.01
	APX	0.738	*p* < 0.01
	CAT	0.830	*p* < 0.01
	GPX	0.624	*p* < 0.01
	AOX	0.796	*p* < 0.01

^1^ 300 and 600 represent two treatment conditions of maximum photosynthetically-active radiation intensity (in µmol·m^–2^·s^–1^), reached at 12:00 h.
